# Restoring and attributing ancient texts using deep neural networks

**DOI:** 10.1038/s41586-022-04448-z

**Published:** 2022-03-09

**Authors:** Yannis Assael, Thea Sommerschield, Brendan Shillingford, Mahyar Bordbar, John Pavlopoulos, Marita Chatzipanagiotou, Ion Androutsopoulos, Jonathan Prag, Nando de Freitas

**Affiliations:** 1 DeepMind, London, UK; 2 Department of Humanities, Ca’ Foscari University of Venice, Venice, Italy; 3 Center for Hellenic Studies, Harvard University, Washington, DC USA; 4 Department of Informatics, Athens University of Economics and Business, Athens, Greece; 5 Faculty of Classics, University of Oxford, Oxford, UK

**Keywords:** Computer science, History, Archaeology

## Abstract

Ancient history relies on disciplines such as epigraphy—the study of inscribed texts known as inscriptions—for evidence of the thought, language, society and history of past civilizations^1^. However, over the centuries, many inscriptions have been damaged to the point of illegibility, transported far from their original location and their date of writing is steeped in uncertainty. Here we present Ithaca, a deep neural network for the textual restoration, geographical attribution and chronological attribution of ancient Greek inscriptions. Ithaca is designed to assist and expand the historian’s workflow. The architecture of Ithaca focuses on collaboration, decision support and interpretability. While Ithaca alone achieves 62% accuracy when restoring damaged texts, the use of Ithaca by historians improved their accuracy from 25% to 72%, confirming the synergistic effect of this research tool. Ithaca can attribute inscriptions to their original location with an accuracy of 71% and can date them to less than 30 years of their ground-truth ranges, redating key texts of Classical Athens and contributing to topical debates in ancient history. This research shows how models such as Ithaca can unlock the cooperative potential between artificial intelligence and historians, transformationally impacting the way that we study and write about one of the most important periods in human history.

## Abstract (web summary)

Ithaca—a deep neural network for textual restoration, geographical attribution and dating of ancient Greek inscriptions—collaboratively aids historians’ study of damaged texts.

## Main

**Fig. 1:**
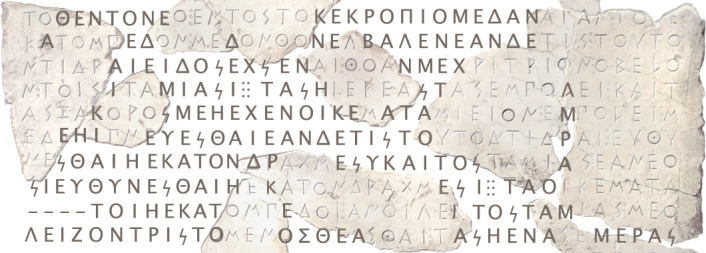
Restoration of a damaged inscription. This inscription (*Inscriptiones Graecae*, volume 1, edition 3, document 4, face B (*IG* I^3^ 4B)) records a decree concerning the Acropolis of Athens and dates to 485/4 bc. Marsyas, Epigraphic Museum, WikiMedia CC BY 2.5.

Epigraphy is the study of texts—inscriptions—written directly on durable materials (stone, pottery, metal) by individuals, groups and institutions of the ancient world^2,3^. Thousands of inscriptions have survived to our time, but many have been damaged over the centuries and their texts are now fragmentary. Inscriptions may also be moved or trafficked far from their original location^4^, and radiocarbon dating is unusable owing to the inorganic nature of most inscribed supports. Specialist epigraphers must then reconstruct the missing text, a process known as text restoration (Fig. 1), and establish the original place and date of writing, tasks known as geographical attribution and chronological attribution, respectively^5^. These three tasks are crucial steps towards placing an inscription both in history and within the world of the people who wrote and read it^6,7^. However, these tasks are non-trivial, and traditional methods in epigraphy involve highly complex, time-consuming and specialized workflows.

When restoring damaged inscriptions, epigraphers rely on accessing vast repositories of information to find textual and contextual parallels^8^. These repositories primarily consist of a researcher’s mnemonic repertoire of parallels and, more recently, of digital corpora for performing ‘string matching’ searches. However, differences in the search query can exclude or obfuscate relevant results, and it is almost impossible to estimate the true probability distribution of possible restorations. Attributing an inscription is equally problematic—if it was moved, or if useful internal dating elements are missing, historians must find alternative criteria to attribute the place and date of writing (such as letterforms, dialects)^9^. Inevitably, a high level of generalization is often involved (chronological attribution intervals can be very long).

## Deep learning for epigraphy

Here we overcome the constraints of current epigraphic methods by using state-of-the-art machine learning research. Inspired by biological neural networks, deep neural networks can discover and harness intricate statistical patterns in vast quantities of data^10^. Recent increases in computational power have enabled these models to tackle challenges of growing sophistication in many fields^11–14^, including the study of ancient languages^15–18^.

We present Ithaca, a deep neural network architecture trained to simultaneously perform the tasks of textual restoration, geographical attribution and chronological attribution. Ithaca, which was named after the Greek island that eluded the hero Odysseus’ homecoming, was trained on inscriptions written in the ancient Greek language and across the ancient Mediterranean world between the seventh century bc and the fifth century ad. This choice was due to two main reasons. First, the variability of contents and context of the Greek epigraphic record, which makes it an excellent challenge for language processing; and second, the availability of digitized corpora for ancient Greek, an essential resource for training machine learning models.

### Working with Greek inscriptions

To train Ithaca, we developed a pipeline to retrieve the unprocessed Packard Humanities Institute (PHI)^19,20^ dataset, which consists of the transcribed texts of 178,551 inscriptions. This process required rendering the text machine-actionable, normalizing epigraphic notations, reducing noise and efficiently handling all irregularities. Each PHI inscription is assigned a unique numerical ID, and is labelled with metadata relating to the place and time of writing. PHI lists a total of 84 ancient regions; whereas the chronological information is noted in a wide variety of formats, varying from historical eras to precise year intervals, written in several languages, lacking in standardized notation and often using fuzzy wording^21^. After crafting an extended ruleset to process and filter the data (Methods), the resulting dataset I.PHI is to our knowledge the largest multitask dataset of machine-actionable epigraphical text, containing 78,608 inscriptions.

### Ithaca is a model for epigraphic tasks

The architecture of Ithaca was carefully tailored to each of the three epigraphic tasks, meaningfully handling long-term context information and producing interpretable outputs to enhance the potential for human–machine cooperation. To begin, contextual information is captured more comprehensively by representing the inputs as words; however, parts of words could have been lost over the centuries. To address this challenge, we process the input text as character and word representations jointly, representing damaged, missing or unknown words with a special symbol ‘[unk]’.

**Fig. 2:**
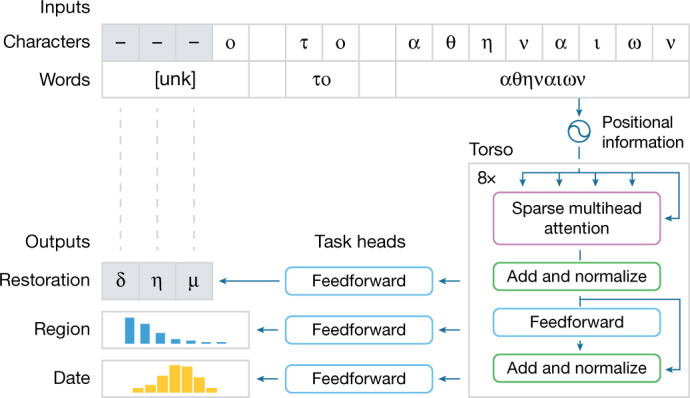
Ithaca’s architecture processing the phrase ‘δήμο το αθηναίων’ (‘the people of Athens’). The first three characters of the phrase were hidden and their restoration is proposed. In tandem, Ithaca also predicts the inscription’s region and date.

Next, to enable large-scale processing, Ithaca’s torso is based on a neural network architecture called the transformer^22^, which uses an attention mechanism to weigh the influence of different parts of the input (such as characters, words) on the model’s decision-making process. The attention mechanism is informed of the position of each part of the input text by concatenating the input character and word representations with their sequential positional information. Ithaca’s torso consists of stacked transformer blocks: each block outputs a sequence of processed representations of which the length is equal to the number of input characters, and the output of each block becomes the input of the next. The final output of the torso is passed to three different task heads that handle restoration, geographical attribution and chronological attribution, respectively. Each head consists of a shallow feedforward neural network, specifically trained for each task. In the example shown in Fig. 2, the restoration head predicts the three missing characters; the geographical attribution head classifies the inscription among 84 regions; and the chronological attribution head dates it to between 800 bc and ad 800.

### Interpreting the outputs

**Fig. 3:**
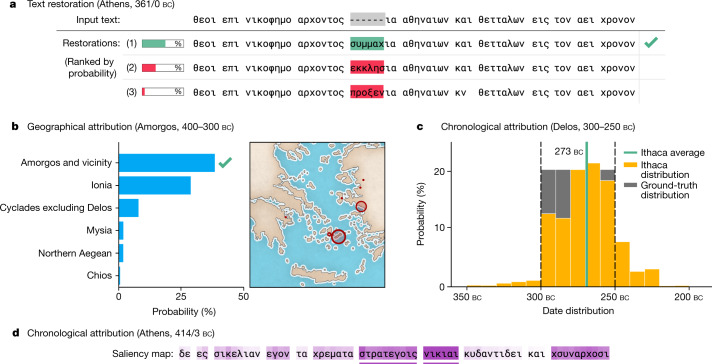
Ithaca’s outputs. **a**, Restoration predictions for six missing characters (dashes) in an Athenian inscription (*IG* II² 116). The top restoration, in green, is correct (συμμαχία, ‘alliance’). Note how the following hypotheses (ἐκκλησία, ‘assembly’; and προξενία, ‘treaty between state and foreigner’), highlighted in red, typically occur in Athenian political decrees^23^, revealing Ithaca’s receptivity to context. **b**, Geographical attribution of an inscription from Amorgos (*IG* XII 7, 2). Ithaca’s top prediction is correct, and the closest predictions are neighbouring regions. **c**, Date distribution for an inscription from Delos (*IG* XI 4, 579). The ground-truth date interval 300–250 bc is shown in grey; Ithaca’s predicted distribution is shown in yellow and has a mean at 273 bc (green). Ithaca’s predictions show a higher confidence for the interval’s higher date margin, therefore potentially narrowing the broad ground-truth dating bracket. **d**, Chronological attribution saliency map for an Athenian inscription (*IG* I³ 371). The colour intensity illustrates the importance of each input. Ithaca focuses on the personal name (Νικίας, ‘Nikias’) and the Greek commanders’ rank (στρατεγοίς, ‘generals’). Nikias had a key role in the great Athenian expedition to Sicily^24–26^, the historical event to which this very inscription pertains. Ithaca dates the inscription to 413 bc, matching the exact range proposed by historians (414–413 bc).

Our intention was to maximize the collaborative potential between historians and deep learning. Ithaca’s architecture was therefore designed to provide intelligible outputs, while featuring multiple visualization methods to augment the interpretability of the model’s predictive hypotheses. For the task of restoration, instead of providing historians with a single restoration hypothesis, Ithaca offers a set of the top 20 decoded predictions ranked by probability (Fig. 3a). This first visualization facilitates the pairing of Ithaca’s suggestions with historians’ contextual knowledge, therefore assisting human decision-making. This is complemented by saliency maps, a method used to identify which unique input features contributed the most to the model’s predictions, for both the restoration and attribution tasks (Fig. 3d and Extended Data Fig. 5a).

For the geographical attribution task, Ithaca classifies the input text among 84 regions, and the ranked list of possible region predictions is visually implemented with both a map and a bar chart (Fig. 3b). Finally, to expand interpretability for the chronological attribution task, instead of outputting a single date value, we predict a categorical distribution over dates (Fig. 3c). By so doing, Ithaca can handle ground-truth labels more effectively, as the labels correspond to date intervals. More precisely, Ithaca discretizes all dates between 800 bc and ad 800 into 10-year bins, resulting in 160 decades. For example, the date range 300–250 bc is represented as 5 decades of equal 20% probability, whereas an inscription dated to 305 bc would be assigned to the single-decade-bin 300–310 bc with 100% probability.

## Experimental evaluation

To compare performance in the three epigraphic tasks, we use four methods. First, we evaluate the difficulty of the restoration task by assigning two evaluators with epigraphical expertise (‘ancient historian’) a set of damaged inscriptions to restore, using the training set to search for textual parallels. Second, we provide the human experts with a ranked list of Ithaca’s top 20 restoration hypotheses to inform their predictions (‘ancient historian and Ithaca’), therefore assessing the true impact of our work as a cooperative research aid. Third, as a computational baseline we reimplement our previous work Pythia^15^—a sequence-to-sequence recurrent neural network for the task of ancient-text restoration. Finally, for the attribution tasks, we introduce an ablation of the epigrapher’s workflow, the ‘onomastics’ baseline: annotators were tasked with attributing a set of texts, exclusively using the known distribution of Greek personal names across time and space to infer geographical and chronological indicia^27^.

We introduce the following metrics to measure each method’s performance. For restoration, to obviate the lack of ground truths in damaged inscriptions, we artificially hide 1 to 10 characters of undamaged input text and treat the original sequences as the target. The first metric used is the character error rate (CER), which counts the normalized differences between the top predicted restoration sequence and the target sequence. Furthermore, we use top-*k* accuracy to measure whether the correct restoration or region label for geographical attribution is among the top *k* predictions, therefore quantifying Ithaca’s potential as an assistive tool. For chronological attribution, we use a distance metric (Methods) to measure the distance in years from the predictive distribution’s mean and the ground-truth interval, the latter being defined by a minimum and a maximum date.

As shown in Table 1, for the task of restoration, Ithaca consistently outperforms the competing methods, scoring a 26.3% CER and 61.8% top 1 accuracy. Specifically, our model achieves a 2.2× lower (that is, better) CER compared with human experts, whereas Ithaca’s top 20 predictions achieve a 1.5× improved performance compared with Pythia, with an accuracy of 78.3%. Notably, when pairing historians with Ithaca (ancient historian and Ithaca), human experts achieve an 18.3% CER and 71.7% top 1 accuracy, therefore demonstrating a considerable 3.2× and 2.8× improvement compared with their original CER and top 1 scores. Regarding the attribution to regions, Ithaca has 70.8% top 1 and 82.1% top 3 predictive accuracy. Finally, for chronological attribution, whereas the onomastics human baseline predictions are within an average of 144.4 and median of 94.5 years from the ground-truth date intervals, Ithaca’s predictions, based on the totality of texts, have an average distance of 29.3 years from the target dating brackets, with a median distance of only 3 years.

**Table 1:** Experimental results

	Restoration			Region		Date
Method	CER (%)	Top 1 (%)	Top 20 (%)	Top 1 (%)	Top 3 (%)	Years
Ancient historian and Ithaca	**18.3**	**71.7**				
Ithaca	26.3	61.8	78.3	**70.8**	**82.1**	**29.3**
Pythia	47.0	32.6	53.9			
Ancient historian	59.6	25.3				
Onomastics				21.2	26.5	144.4

Evaluating methods for text restoration, geographical attribution (region) and chronological attribution (date) on I.PHI’s test set of *n* = 7,811 inscriptions. For ‘CER’ and ‘years’, lower scores are better. For ‘top 1’, ‘top 3’ and ‘top 20’, higher scores are better. For each metric, the best performing method is in bold.

## Contributing to historical debates

Our experimental evaluation effectively demonstrates Ithaca’s impact on the study of inscriptions, and their consequent value as historical evidence. First, Ithaca can discover epigraphic patterns on an unprecedented scale and in unparalleled detail, harnessing substantial quantities of epigraphic data (I.PHI) to achieve the high performance observed in all three epigraphic tasks. Moreover, whereas Ithaca may have outperformed historians in the first baseline, the combination of a historian’s own (contextual) knowledge alongside Ithaca’s assistive input resulted in an even greater improvement over the model’s performance. This collaborative potential is augmented by Ithaca’s design decisions, and by the different visualization aids increasing the interpretability of outputs, therefore enabling historians to evaluate multiple hypotheses. As a consequence, Ithaca could help historians narrow the wide or vague date brackets they are sometimes forced to resort to, by helping increase precision and establish relative datings for historical events, and even contributing to current methodological debates in ancient history.

Indeed, to demonstrate Ithaca’s creative potential, we applied our model to a contemporary dispute concerning the dating of a group of inscriptions whose interpretation is central to the political history of classical Athens. Historians disagree on whether these decrees should pre- or post-date 446/5 bc depending on the (dis)belief in using specific letterforms as dating criteria (the three-bar sigma dating convention)^28^. In recent years, the validity of this dating convention was called into question^29^—the dates of many decrees have been pushed to the 420s bc, therefore profoundly influencing our understanding of Athenian imperialism^30^.

This group of disputed Athenian decrees exists in our dataset: their dating labels follow the conventional ‘higher’ dates (pre-446/5 bc). We excluded these texts from the dataset and trained Ithaca on all of the remaining inscriptions. Notably, Ithaca’s predictions for these held-out texts independently align with the most recent dating breakthroughs, therefore overturning the conventional historical reading based on the sigma dating criterion. More specifically, whereas the I.PHI labels are on average 27 years off the ‘lower’ dating proposed by modern re-evaluations, Ithaca’s predictions are on average only 5 years off the newly proposed ground truths.

This example eloquently illustrates how models such as Ithaca can contribute to key methodological debates on the chronological reorganization of Athenian imperialism, one of the most important moments in Greek history. In no instance do Ithaca’s predictions for this group of inscriptions exceed 433 bc: Ithaca’s average predicted date for all of these decrees is 421 bc. Historians may now use Ithaca’s interpretability-augmenting aids (such as saliency maps) to examine these predictions further and bring more clarity to Athenian history.

## Conclusions

Ithaca is to our knowledge the first epigraphic restoration and attribution model of its kind. By substantially improving the accuracy and speed of the epigrapher’s pipeline, it may assist the restoration and attribution of newly discovered or uncertain inscriptions, transforming their value as historical sources and helping historians to achieve a more holistic understanding of the distribution and nature of epigraphic habits across the ancient world. To achieve this goal, our interdisciplinary team created an open-source and publicly available interface (https://ithaca.deepmind.com), enabling historians to use Ithaca for their personal research, while facilitating its development for further applications.

In fact, the methods introduced in this research apply to all disciplines dealing with ancient text (papyrology, numismatics, codicology), to any language (ancient or modern), also integrating additional metadata (inscription images, stylometrics). Furthermore, Ithaca’s quintessentially interactive nature as a cooperative research aid lends itself as an effective set-up for future machine learning research by adding humans into the training loop.

In conclusion, the transformational impact of this work lies in delivering state-of-the-art research aids that extend the scope of ancient history and the humanities.

## Methods

### Previous work

In recent years, several works have proposed traditional machine learning approaches to the study of ancient texts. This body of work has focused on optical character recognition and visual analysis^31–34^, writer identification^35–37^ and text analysis^38–44^, stylometrics^45^ and document dating^46^. It is only very recently that scholarship has begun to use deep learning and neural networks for optical character recognition^47–55^, text analysis^56^, machine translation of ancient texts^57–59^, authorship attribution^60,61^ and deciphering ancient languages^62,63^, and been applied to study the form and style of epigraphic monuments^64^.

The closest work to Ithaca is our 2019 research on ancient text restoration: Pythia^15^. Pythia was to our knowledge the first ancient text restoration model to use deep neural networks, and was followed by blank language models^18^, Babylonian^65^ and Korean text translation and restoration^17^, Latin BERT for language modelling, part-of-speech tagging, word sense disambiguation and word similarity^16^, and the classification of Cuneiform tablets by period^66^.

Ithaca is to our knowledge the first model to tackle the three central tasks in the epigrapher’s workflow holistically. Not only does it advance the previous state-of-the-art set by Pythia, but it also uses deep learning for geographical and chronological attribution for the very first time and on an unprecedented scale. Ithaca offers interpretable outputs, showcasing the rising importance of cooperation between human experts and machine learning^67^—as exemplified by our experimental evaluation.

Most importantly, this work shows how matching human experts with deep learning architectures to tackle tasks collaboratively can surpass the individual (unaided) performance of both humans and model on the same tasks. Indeed, recent medical research^68,69^ further confirms the importance of hybrid architectures in addressing real-world problems. The present work makes human expert interaction possible by visualizing the output probability distributions for all tasks using multiple charts and maps, and augmenting their interpretability by means of saliency maps. It is our hope that this work may set a new standard for the field of digital epigraphy, by using advanced deep learning architectures to support the work of ancient historians.

### Generating the I.PHI corpus

When restoring damaged inscriptions, epigraphers conjecture the total number of missing characters based on grammatical and syntactical considerations, and on the reconstructed physical form of the text^5^. Conjectured missing characters that cannot be restored are conventionally marked with periods or hyphens, one hyphen equating to one missing character. Moreover, PHI presents interpretive transcriptions of the texts (including capitalization, punctuation, word division, lower-case letter conversion).

**Extended Data Fig. 1:**
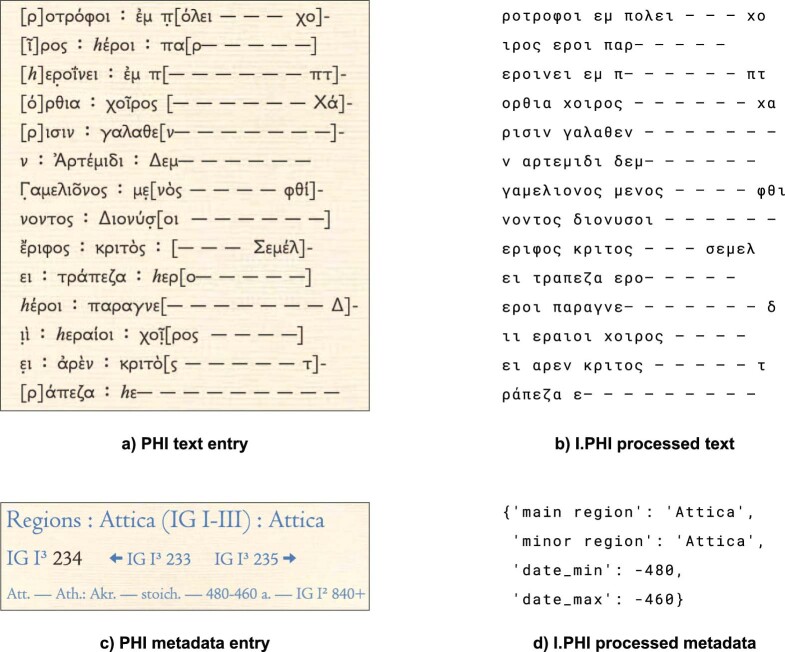
Raw and processed PHI inscription, text and metadata. A fragmentary early-fifth century sacrificial calendar from the Acropolis of Athens (*IG* I^3^ 234), face A, lines 10-23. In (**a**) transcription of the inscription text as it currently appears in PHI; (**b**) the same text’s processed rendition in I.PHI; (**c**) the unprocessed metadata of this inscription as it currently appears in the PHI dataset; (**d**) the processed metadata rendition in I.PHI.

Thus, moving from the PHI dataset, we substantially expand the ruleset for filtering human annotations previously conceived for Pythia, rendering the text machine-actionable. We removed 9,441 duplicate texts and filtered out all inscriptions under 50 characters in length, whereas, in Pythia’s dataset, we had excluded all texts with fewer than 100 characters. To increase the amount of available text, we retained the supplements proposed by epigraphers (conventionally added between square brackets), and we matched the number of unrestored characters with an equal number of ‘–’ symbols, as is commonly done by epigraphers (Extended Data Fig. 1).

**Extended Data Fig. 2:**
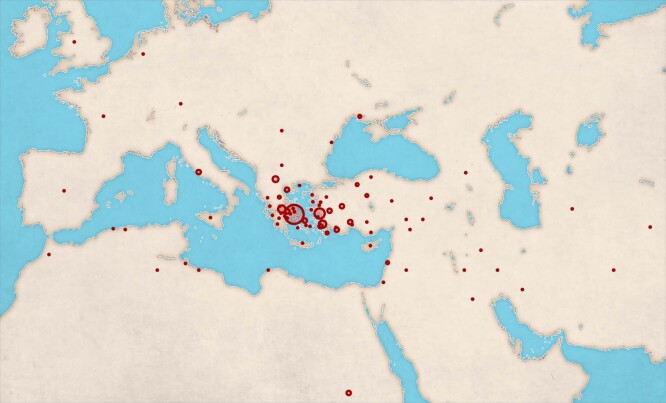
Geographical distribution of Greek inscriptions in I.PHI. Each red circle represents a region across the ancient Mediterranean world (84 in total), the circle size is directly proportional to the number of inscriptions found in that region (total inscriptions in I.PHI n = 78,608).

Each PHI inscription is assigned to a region of the ancient Mediterranean world (Extended Data Fig. 2), and includes an additional metadata string referring to the date proposed by epigraphers for the text (Extended Data Fig. 1). The chronological information is noted in a variety of formats (historical eras, precise year intervals); in several languages (including Latin); ranging before (bce) and after (ce) the Common Era; lacking in standardized notation (‘early’, ‘first half’, ‘1st half’, ‘beginning’, ‘beg.’) and often using fuzzy wording (‘late 7th/6th ac.’, ‘ca. 100 a.?’, ‘bef. 64 ad’). After crafting an extended ruleset, we succeeded in generating well-defined date intervals for 60% of all PHI inscriptions, as the chronological metadata of the remaining 40% is either missing or unprocessable. The resulting I.PHI dataset contains 1.93× more inscriptions than the previous Pythia’s dataset. The texts of which the numerical PHI identifier (PHI ID) ended in 3 or 4 were held out and used as test and validation sets, respectively (Extended Data Table 1).

### Ithaca architecture

#### Inputs

For each inscription, the input of the model consists of (1) a sequence of character embeddings (real-valued vectors, each representing the character of the alphabet that occurs at the corresponding position of the inscription); (2) an equally long sequence of word embeddings (real-valued vectors, each representing the vocabulary word at the corresponding character position of the inscription; Fig. 2); and (3) positional embeddings (also real-valued vectors, each representing a position of the input sequence). The first two kinds of embeddings are randomly initialized and learned when training Ithaca (via backpropagation). The positional embeddings are also trainable and they are initialized with a separate sinusoidal function per dimension^22^ to maintain a symmetrical distance between neighbouring steps and smoothly decay over the maximum length of 768 characters. Our vocabulary includes every word appearing more than 10 times in I.PHI (35,884 words), while damaged or ‘unknown’ (under-represented) words are rendered with an ‘[unk]’ symbol. The joint use of character and word embeddings enables the architecture of Ithaca to be both character- and context-aware^70–72^. Finally, the input sequence is padded with a start-of-sentence character ‘<’.

#### Torso

The three input sequences are combined by concatenating the different embeddings per-character position and the resulting sequence is fed through the torso of the model. The architecture of Ithaca’s torso consists of eight stacked transformer decoder blocks, inspired by the large-scale transformer model BigBird^73^. Every block uses four sparse attention heads (using global, local and random attention mechanisms), which reduce the context-length dependency from quadratic to linear, therefore enabling the model to handle lengthier sequences^73^ compared with classical transformers. Furthermore, the attention mechanism is ‘multi-head’ (Fig. 2) in the sense that it can learn to consider different types of information extracted from the input. For example, different attention heads may be sensitive to particular character sequences, or more perceptive to certain words and phrases with distinctive morphosyntactic or semantic features. Finally, to overcome problems that hinder the stacking of such complicated blocks, each transformer block uses residual connections and layer normalization (shown as ‘add and normalize’ in Fig. 2).

#### Task heads

Ithaca’s torso outputs a sequence whose length is equal to the number of input characters, and each item in this sequence is a 2,048-dimensional embedding vector. Each task head consists of a two-layer feedforward network followed by a softmax function. There are three different task heads, handling region attribution, chronological attribution and restoration respectively. To predict the regions and dates, Ithaca uses the first output embedding (*t* = 1) and passes it on to the two corresponding heads. This arrangement is similar to that of DocBERT^74^ and works better than other pooling methods (such as mean- and max-pooling over the output embeddings) in our experimental evaluation. Finally, for the restoration task, Ithaca uses the remaining output embeddings (*t* > 1) as there is a direct correspondence with the input text characters: for each missing character position, the corresponding output embedding of the torso is fed to the head of the restoration task, which predicts the missing character.

### Data preparation and augmentation

I.PHI may be the first multitask dataset of machine-actionable epigraphical text, but its size is still several orders of magnitude smaller than modern typical language datasets. To avert the risk of overfitting, which is common in large-scale deep neural network architectures, we apply several data augmentation methods, described below, to artificially increase the size of I.PHI’s training set. Our preliminary experimental evaluation found that these methods are crucial in achieving the reported performance. These augmentation methods are applied anew whenever a training inscription is re-encountered in each training epoch.

#### Text clipping

For each inscription, we select an arbitrary section of its text and ignore the remaining text. We implement this by first sampling a segment length between 50 and 768 characters, and then sampling the starting index of the segment. This method helps Ithaca to generalize and improve the handling of partial inputs.

#### Text masking

Forcing the model to rely on contextual information often leads to improvements in prediction. To achieve this in our model, during training, we randomly hide up to half of the input text by replacing sequences of characters sampled from a geometric distribution (*P* = 0.1) with ‘–’. This span masking is intended to replicate the distribution over the length of missing characters estimated from the dataset, and uses the hidden ground-truth characters as target labels for the restoration task.

#### Word deletion

During training, we also delete words from each input text (without replacing them with any special characters in this case) with a 20% probability. Here, the goal is again to increase variability in the training data to improve the model’s ability to generalize over all possible ways in which inscriptions are damaged^75^.

#### Sentence swap

By randomly swapping sentences in the input text with a 25% probability, we generate multiple input–label pairs for the auxiliary task of next-sentence prediction (NSP)^75^ (see below).

### Data circularity

Ithaca’s source dataset (PHI) is a synthesis of generations of scholarly research. Epigraphers typically restore texts and attribute them chronologically through a process of induction. Textual restorations are proposed on the basis of parallels, mediated by wider historical and linguistic knowledge; chronological attributions are proposed partly from archaeological and contextual information, partly from textual form and content, and partly from textual and material parallels. The texts on which Ithaca trains include previous scholarly restorations; and the dates recorded are the product of accumulated scholarly knowledge and induction from archaeological, historical and textual study. This might be thought to imply circularity, but that would be true only if Ithaca were operating in a world of objective data and aiming to offer a single objectively true solution. Rather, Ithaca is an assistive tool aiming to improve on and facilitate a scholarly process of induction, model uncertainty and propose possible solutions for the scholar to consider.

Considering textual restoration, Ithaca avoids the risk of ‘history from square brackets’^76–78^ (assuming any proposed restoration to be ground truth, meaning the accepted consensus, rather than merely one of several hypotheses), because none of Ithaca’s proposed restorations are assumed to be objectively certain—instead, they are presented as plausible suggestions. Furthermore, the inclusion of existing scholarly conjectures within the training set itself does not constitute a form of ‘history from square brackets’, as such conjectures are themselves plausible restorations achieved by a process of induction and considered acceptable by one or more experts, and as such are precisely the sort of result that Ithaca itself aims to generate. The value of Ithaca is indeed its ability to learn from the largest possible dataset of attested and possible texts, making the underlying process of inductive reasoning as powerful as possible, and so generating possible restorations for scholars to evaluate.

As for chronological attribution, the dataset on which Ithaca trains is founded in the past study of multiple elements (such as archaeological provenance, material form, textual content and form). Ithaca in turn learns through close attention to the text alone. The attributions proposed by Ithaca therefore have their basis in the inductive study of a vast textual dataset and its correlation to chronological data that are more broadly derived. Ithaca is therefore able to bring some refinement to those attempts to date the texts through the application of machine learning specifically to the textual patterns in that data. Thus, Ithaca is, in this case, a part of that scholarly process, and no more or less circular in its reasoning than any other scholar.

### Training on epigraphic tasks

For the task of restoration, we use the text-masking augmentation method to mask parts of the input and produce ground truths. We subsequently use a cross-entropy loss to train Ithaca to predict the missing characters. The cross-entropy loss is also used for geographical attribution, using the region metadata as target labels. We further apply label smoothing with a coefficient of 10% to avoid overfitting and to provide historians with a smoother distribution of predicted hypotheses. For the task of chronological attribution, Ithaca discretizes all dates between 800 bc and ad 800 with a bin size of 10 years. This range covers the majority of the PHI dataset entries and encompasses the conventional date range for Greek epigraphy. The processed ground-truth date intervals are discretized into bins of equal probability, forming the target probability distribution. The limitations of discretizing and amalgamating date ranges of different levels of precision based on past scholarship have been noted^79,80^—the scale of data on which Ithaca trains, together with the increased attention to textual patterns (compared with the previous paragraph), at least partially meet that challenge. We then use the Kullback–Leibler divergence to minimize the difference between target and predicted probability distribution (Fig. 3c).

Finally, to allow for better modelling of context, we introduce a next sentence prediction loss, an auxiliary function common to language modelling tasks^81^. During training, we randomly shuffle some of the sentences of the input text, and at the end of each (non-final) sentence (marked by a full stop, ʻ.ʼ) we predict whether the next sentence is in the correct order (valid) or a product of the shuffling augmentation. By deploying the torso’s output embeddings for the full stops, we introduce an additional feedforward network that uses binary cross-entropy to predict the validity of the next sentence whenever a ʻ.ʼ character appears.

Using this setup, Ithaca was trained for a week on 128 Tensor Processing Units (TPU) v4 pods on the Google Cloud Platform. The effective batch size was 8,192 texts and a LAMB optimizer^82^ was used to optimize Ithaca’s parameters with a learning rate of 3 × 10^−4^. Using Bayesian optimization hyperparameter search, the loss functions of each task were combined using the following function:

$$L=3\times {L}_{{\rm{Restoration}}}+2\times {L}_{{\rm{Region}}}+1.25\times {L}_{{\rm{Date}}}+0.01\times {L}_{{\rm{NSP}}}.$$

We do not use a separate masked (token) language modelling loss, which is commonly used when pretraining language models, as it is very similar to the restoration loss, although the latter masks characters instead of tokens.

To obtain Ithaca’s textual restoration predictions, we select a sequence of missing characters to predict and use Beam Search with a beam width of 100. Instead of using a standard sequential Beam Search, we take advantage of Ithaca’s non-autoregressive nature^83–85^, and use a non-sequential one instead. Each beam starts with the prediction scoring the highest confidence^86^, then proceeds iteratively to restore at each time-step the characters of which the certainty is the highest. We found that this version of Beam Search performed substantially better in our evaluation metrics. For region attribution, the outputs are presented as a plot of the top 10 predictions; for chronological attributions, we visualize the model’s predictive distribution over possible date bins. Finally, to reduce the variance of random segment selections, we repeat the process ten times and report results averaged over the iterations.

### Ancient historian baseline

The evaluators for ancient text restoration were two graduate students of ancient history, with 7 years of historical and linguistic training and specializing in Greek history and epigraphic documents. Thus, they can be assumed to be more capable than the ‘average’ ancient historian, but not yet equivalent to (the very small number) of established specialists in the field. The scholars were allowed to use the training set to search for textual ‘parallels’, and made an average of 50 restorations in 2 h.

Although Ithaca can indeed propose restoration hypotheses faster, and model its prediction uncertainty, it cannot make choices on the basis of historical and material context. Thus, the experimental setup cannot be considered to be direct comparison between human historians and machine learning, nor are the evaluators assumed to be a proxy for all historians. Instead, the experiment was intended to measure the difficulty of the task and the potential for cooperative artificial intelligence.

### Onomastics baseline

Greek nomenclature is commonly used by epigraphers as one of several elements to inform their attribution predictions^87^. Inspired by this method in the wider epigraphic workflow, we designed an ‘onomastic’ baseline, of which the predictions are based exclusively on the metadata associated with Greek personal names. Five annotators searched for name(s) appearing in a set of inscriptions in the Lexicon of Greek Personal Names (LGPN), a database recording the geographical and chronological distribution of ancient names^27^, and based their attribution hypotheses on the LGPN’s distribution data. Evaluators were also provided with the inscription’s date or place of writing for the geographical or chronological attribution tasks, respectively.

### Restoration metrics

To evaluate different restoration methods, for every inscription, we predict a sequence of 1–10 contiguous missing characters. These lengths account for 83% of the distribution of missing character lengths in I.PHI, and enable comparisons with both previous work and the human baselines. Note that, thanks to the text-masking augmentation adopted during training, Ithaca could potentially restore up to half of the input text.

Although the number of characters to be predicted reflects the difficulty of the task, the restored sequences in the test sets held out for human evaluation might not necessarily maintain the same distribution of lengths (as they were a subset of the test set). Thus, instead of reporting only the average scores over the entire test set (as done in previous work), we chose to account for these length discrepancies and compute the average scores for each restored sequence length. First, we computed a separate CER for all samples of each length (between 1–10 characters),

$${{\rm{CER}}}_{l}=\frac{1}{{\sum }_{i}^{N}{I}_{{{\rm{len}}}_{i}=l}}\mathop{\sum }\limits_{i}^{N}{I}_{{{\rm{len}}}_{i}=l}\times \frac{{\rm{EditDistance}}({{\rm{pred}}}_{i},{{\rm{target}}}_{i})}{l},$$

where *I* is the indicator function, len_*i*_ denotes the length of the *i*-th sample, *N* is the number of samples, pred_*i*_ is the predicted sequence of missing characters of the *i*-th sample and target_*i*_ the corresponding target sequence. We next calculate the average for all lengths:

$${{\rm{CER}}}_{{\rm{score}}}=\frac{1}{L}\mathop{\sum }\limits_{l}^{L}{{\rm{CER}}}_{l}.$$

where *L* = 10 is the maximum length.

As human annotators annotated only a subset of the test set owing to time constraints, macro-averaging assigns equal importance to all sample lengths to represent the difficulty of the task independently of dataset statistics, and therefore enabling a fair comparison of the methods. Similarly, for accuracy, we first computed a separate accuracy per length, and then the average:

$${{\rm{a}}{\rm{c}}{\rm{c}}{\rm{u}}{\rm{r}}{\rm{a}}{\rm{c}}{\rm{y}}}_{l}=\frac{1}{{\sum }_{i}^{N}{I}_{{{\rm{l}}{\rm{e}}{\rm{n}}}_{i}=l}}\mathop{\sum }\limits_{i}^{N}{I}_{{{\rm{l}}{\rm{e}}{\rm{n}}}_{i}=l}\times {I}_{{{\rm{p}}{\rm{r}}{\rm{e}}{\rm{d}}}_{i}={{\rm{t}}{\rm{a}}{\rm{r}}{\rm{g}}{\rm{e}}{\rm{t}}}_{i}},$$

$${{\rm{accuracy}}}_{{\rm{score}}}=\frac{1}{L}\mathop{\sum }\limits_{l}^{L}{{\rm{accuracy}}}_{l}.$$

### Chronological attribution metric

As our model outputs a predictive distribution in the chronological attribution task, we introduce an interpretable metric to measure the distance in years between a prediction and the ground-truth interval (Fig. 3c). More specifically, we use a distance metric between the mean of the predictive distribution and the target ground-truth interval; the latter is defined by a minimum (gt_min_) and a maximum (gt_max_) date in years:

$${\rm{Years}}=\{\begin{array}{cc}0, & {{\rm{if\; gt}}}_{{\rm{\max }}}\ge {{\rm{pred}}}_{{\rm{avg}}}\ge {{\rm{gt}}}_{{\rm{\min }}}\\ |{{\rm{pred}}}_{{\rm{avg}}}-{{\rm{gt}}}_{{\rm{\max }}}|, & {{\rm{if\; pred}}}_{{\rm{avg}}} > {{\rm{gt}}}_{{\rm{\max }}}\\ |{{\rm{pred}}}_{{\rm{avg}}}-{{\rm{gt}}}_{{\rm{\min }}}|, & {{\rm{if\; pred}}}_{{\rm{avg}}} < {{\rm{gt}}}_{{\rm{\min }}}\end{array}.$$

### Model selection

The final model was obtained by storing the best-performing model on the validation set by using a combined metric that sums the accuracy for textual restoration and geographical attribution, and the distance in years divided by 100 for chronological attribution to make the magnitude comparable. The extensive computational resources required to train our model made the Pareto frontier computation infeasible.

### Chronological attribution results

**Extended Data Fig. 3:**
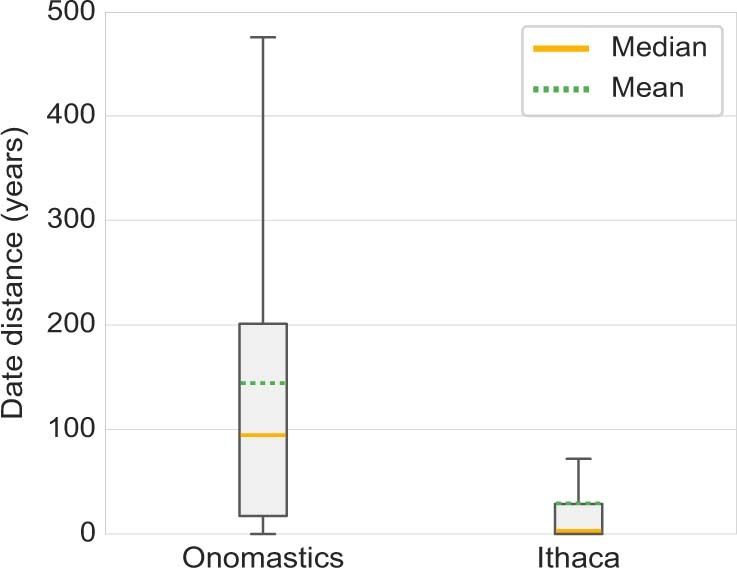
Comparison between Ithaca and the onomastics baseline’s chronological predictions. The box plot shows the median and the mean distance between the predicted date and the ground-truth time interval, measured in years using the chronological distance metric (see Methods). In this plot, the bounds of the boxes are defined by the first and the third quartiles, and the whiskers by the minimum and maximum values. Ithaca’s mean distance is 2.2x lower than that of the onomastics baseline. Ithaca’s average prediction loss was 29.3 years from the ground-truth interval, while the median prediction loss was only 3 years. The onomastics baseline consists of n = 142 attributions provided by the human annotators.

Ithaca’s predictions are 5× closer to ground truths than those recorded in the onomastics baseline (144.4 years). More specifically, Ithaca’s average date prediction is within 28.7 years of the ground-truth date interval, and the median is only 3 years. The results are shown in detail in Extended Data Fig. 3.

### Restoring full texts with Ithaca

**Extended Data Fig. 4:**
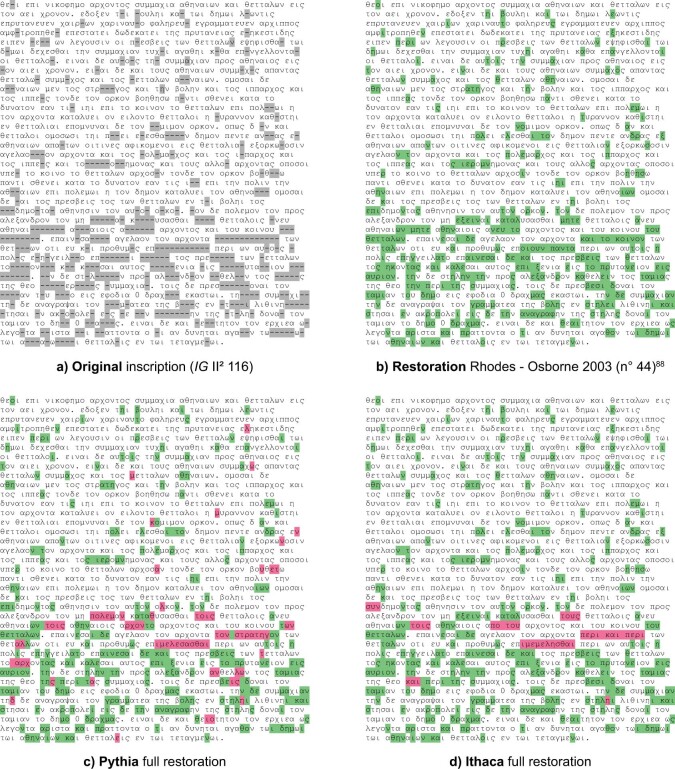
Restoration performance comparison. (**a**) The original inscription (*IG* II² 116) has 378 missing characters. (**b**) The restorations of the missing characters proposed in the authoritative edition by Rhodes - Osborne 2003 for this text^88^, and which we use as ground truths in our evaluation. (**c**) Pythia’s restoration shows 74 mismatches with the Rhodes-Osborne edition, while (**d**) Ithaca’s shows only 45. Correct restorations are highlighted in green, incorrect ones in red.

To overcome memory constraints and length limitations for long inscriptions (>768 characters), Ithaca can be applied iteratively to restore all missing text in a damaged inscription. We experimented with this option on inscription *IG* II² 116, which is missing 378 characters, and compared Ithaca’s predictions with those of our previous work Pythia on the same text, using the authoritative edition published by Rhodes and Osborne as ground truths^88^. The models’ correct restorations are highlighted in green (Extended Data Fig. 4), and the erroneous ones in red. In a real-world scenario, both Ithaca and Pythia would provide a ranked set of 20 restoration hypotheses. The comparison in performance between Pythia and Ithaca is stark (74 versus 45 mistakes): moreover, in all cases in which the restoration is in red, the ground-truth sequence existed within the beam of Ithaca’s top 20 hypotheses.

### Geographical attribution of Delphic inscriptions

Epigraphers determine the original location where an inscription was written by examining the personal names, local or regional dialectal varieties, and idiosyncratic lexicon or style of an inscription. Moving from this methodological premise, and to discover underlying patterns in Ithaca’s geographical predictions, we compute statistics to track the words that appear most frequently in texts whose region Ithaca predicts correctly. Thus, for each word of the test set, we compute an average accuracy and a frequency of appearance. This visualization is intended to evaluate whether the occurrence of particular words could be correlated to the model’s geographical attributions.

The most frequent words that appear in texts with high prediction accuracy clustered primarily in inscriptions from the region of Delphi, and pertained to the epigraphic genre of ‘manumission inscriptions’ (Extended Data Table 2 for an example). Ancient Greek society depended heavily on unfree labour, but slaves could be freed through a process known as ‘manumission’, which was publicly documented and certified by inscriptions^89,90^. Over 1,000 such texts dating between around 201 bc and ad 100 have been found in Delphi^91,92^. The words appearing in Ithaca’s accuracy statistics are identified as typical of these manumission texts, which are in turn distinctive of this region (for example, ἐπίστευσε, άποδμενος, καταδουλισμωι, βεβαιωτήρ, ωνάν): these words could therefore be underpinning the correct attribution predictions (a detailed example is offered in Extended Data Table 2). Further study can now be dedicated to investigating stylized manumissions as distinctive of Delphi.

**Extended Data Fig. 5:**
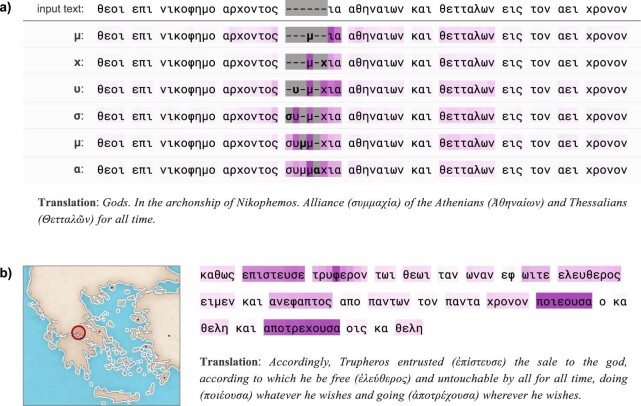
Restoration and geographical attribution saliency maps. (**a**) The decree (*IG* II² 116) from the Acropolis of Athens recording an alliance between the Athenians and the Thessalian federation (360/1 bc). At each step of the restoration of the missing word “alliance” (συμμαχία), Ithaca is clearly attending to the contextually important words “Athenians” (ʻAθηναίων) and “Thessalians” (Θετταλων). (**b**) The manumission inscription (*BCH* 66/67 (1942/3) 82,9) is correctly attributed to the Delphi region (left), and the generated saliency map (right) highlights words correlated to high accuracy predictions from the word statistics table.

To further assess the impact of Ithaca’s output visualization techniques in a real-world scenario, we also analysed the saliency maps for geographical attribution of the manumission inscriptions. Indeed, the saliency maps for the Delphic inscription *BCH* 66/67 (1942/3) 82,9, for example, highlight words typically found in manumission texts and which also appear in Ithaca’s word statistics: these words (ἐπίστευσε, ἐλευθερος, ποιέουσα, ἀποτρέχουσα) have the most important role in the geographical attribution of the inscription, while also betraying the text’s genre as a typical slave manumission inscription (Extended Data Fig. 5b).

### Redating disputed Athenian decrees

In the absence of helpful internal evidence of a text’s date (for example, the mention of known historical figures^93^), epigraphers typically derive an approximate date on the basis of a text’s content, letterforms and grammatical criteria. For example, one of the most notorious methodological debates in epigraphy concerns the ‘three-bar sigma’ dating convention, which holds that no Athenian public document containing the three-bar sigma letter (ϟ) could be dated after the year 446/5 bc, when the letter was supplanted by the four-bar sigma (Σ). On the basis of this chronological benchmark, a group of inscriptions whose interpretation is central to the political history of Classical Athens, and which feature the earlier letter ϟ, were dated to pre-446/5 bc by many authoritative corpora^28^, ^94^. This set of decrees exists in the PHI dataset (Extended Data Table 3), and their dating labels follow the conventional ‘higher’ dating of the three-bar sigma criterion.

However, this orthodox dating system soon proved to be problematic: the high dates proposed for these decrees did not agree with contemporary literary accounts reporting on Athenian imperialist policies. Few historians contested the validity of the sigma criterion^29,95^, but in 1990 photo-enhancement and laser scanning confirmed the down-dating of an inscription featuring the three-bar sigma (the Egesta decree, *IG* I^3^ 11) from 458 to 418 bc^96^. Over the following decade, the sigma’s traditional cut-off date was revisited, and the dates of other decrees were also pushed back^28,97^.

**Extended Data Fig. 6:**
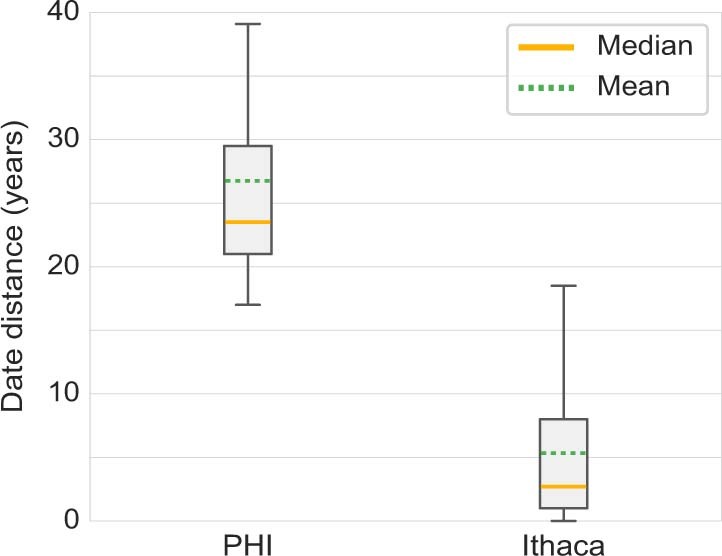
PHI vs. Ithaca’s dating distance in years for disputed Athenian decrees. The box plot shows the median and the mean of the distribution, the bounds of the boxes are defined by the first and the third quartiles and the whiskers by the minimum and maximum values of n = 21 inscriptions. Ithaca’s chronological predictions (average distance of 5 years from the modern “lower” ground truth) compared to PHI meta-data for time intervals (older estimates, average distance of 27 years from the modern ground truth). Lower distance in years is better. Exploiting the features of our full dataset, Ithaca’s predictions are better and closer to modern re-evaluations compared to the original PHI ground-truth dates. The latter reflect the dates assigned by the published editions which PHI is reporting, and which almost all reflect the old three-bar sigma dating. We refer the reader to Extended Data Table 3 for detailed results.

**Extended Data Fig. 7:**
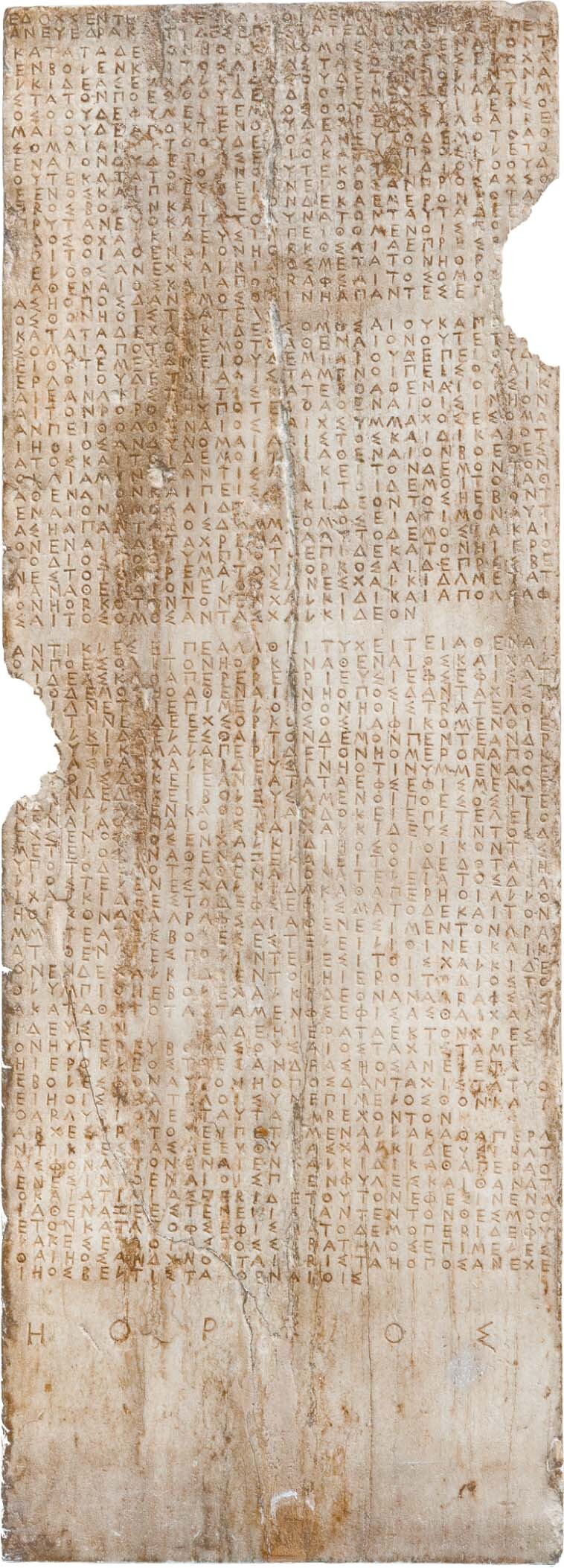
Chalcis decree (*IG* I^3^ 40). The inscription records an oath of allegiance sworn by the city of Chalcis to Athens. It has been traditionally dated to 446/5 bc based on the 3-bar sigma criterion^28^, but was more recently redated to 424/3 bc^99^. Photograph by kind concession of the Acropolis Museum. Acrop. 6509 © Acropolis Museum (photo: Socratis Mavrommatis).

Ithaca’s predictions for this set of disputed inscriptions independently align with the most recent dating breakthroughs (Extended Data Fig. 6). For example, the (in)famous Chalcis decree (*IG* I^3^ 40; Extended Data Fig. 7), which records an oath of allegiance sworn by the city of Chalcis to Athens^98^ and traditionally dated to 446/5 bc^28^, is attributed by Ithaca to 420 bc, therefore concurring with the lower dating hypothesis of 424/3 bc proposed by more recent scholarship^99^. Perhaps the most compelling example of Ithaca’s prediction independently aligning with a lower dating hypothesis is the decree of Kleinias (*IG* I^3^ 34)^100^, regulating the collection of tribute across the Athenian empire. The sigma dating system would assign the inscription to 448/7 bc^28^, but scholars have recently challenged this orthodoxy and proposed the earlier date of 425/4 bc^101^. Ithaca’s prediction agrees precisely with the latter, dating the famous decree to 424 bc.

Ithaca has re-dated a number of these key inscriptions with striking accuracy (Extended Data Table 3). Although it may seem slight, this 40/30-year chronological reorganization has considerable implications for our grasp of Athenian imperial behaviour, leading historians to a more profound understanding of one of the most momentous periods of ancient history^28,97^. The fact that Ithaca was trained on the largest available dataset of Greek epigraphic texts makes it possible to challenge or overcome individual biases or, indeed, errors in the existing academic tradition, notwithstanding the fact that the dataset in question is originally based on the accumulated academic tradition.

### Reporting summary

Further information on research design is available in the Nature Research Reporting Summary linked to this paper.

## Online content

Any methods, additional references, Nature Research reporting summaries, source data, extended data, supplementary information, acknowledgements, peer review information; details of author contributions and competing interests; and statements of data and code availability are available at 10.1038/s41586-022-04448-z.

### Supplementary information

Reporting Summary

## Figures and Tables

**Extended Data Table 1:**
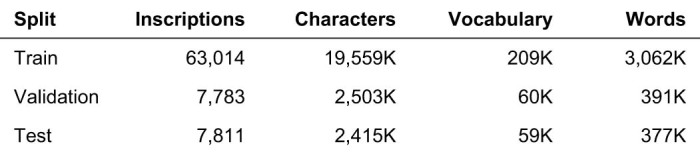
Dataset statistics for the size of the I.PHI corpus

**Extended Data Table 2:**
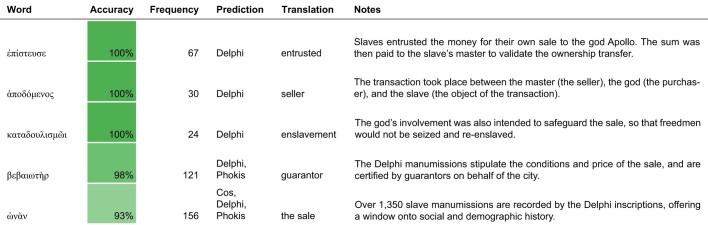
Word statistics for geographical attribution

To discover underlying patterns in Ithaca’s predictions, we compute statistics to track the words that appear most frequently (“frequency”) in texts whose region Ithaca predicts correctly (“accuracy”). For each word of the test set, we compute an average accuracy, and a frequency of appearance. This visualization is intended to evaluate whether the occurrence of particular words could be correlated to the model’s geographical attributions.

**Extended Data Table 3:**
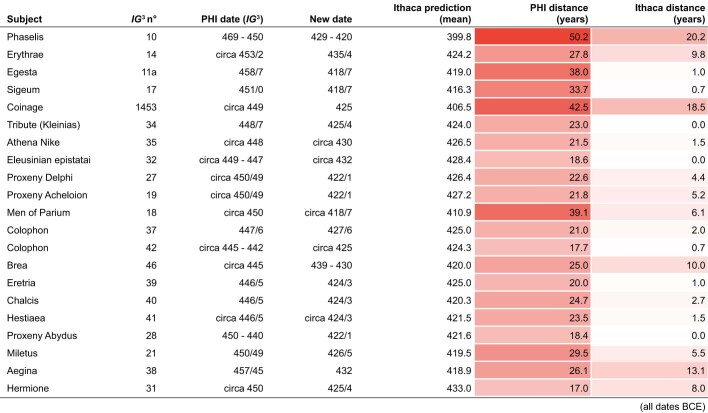
Downdating Athenian decrees with Ithaca

List of disputed Classical Athenian decrees (including their *IG*^3^ edition number), their dates as listed in PHI (which follow the conventional dates proposed by Meiggs - Lewis 1969^103^ and correspond to the dates in the *IG*^3^ editions of the decrees) based on the conventional ‘three-bar-sigma’ dating criterion, and their recent dating re-evaluations^28^. Ithaca’s prediction mean is listed in column 5. The last two columns represent the distance (in years) of the PHI dates and Ithaca’s predictions from the recent dating re-evaluations. The colour intensity reflects the distance in years, with stronger intensity reflecting a farther distance. As can be seen, Ithaca’s predictions result in an average distance of 5 years, which is 22 years closer to the re-evaluated dates, compared to PHI’s conventional dates.

PHI IDs of the inscriptions excluded from training: 10, 11, 14, 17, 18, 19, 27, 28, 32, 34, 37, 39, 40, 41, 42, 46, 1682; additional PHI IDs for new editions, newly discovered or published sections and doubles of the decrees: 293752, 294468, 229647, 291317, 232697, 293754, 1675, 1676, 1677, 1678, 1679, 1680, 1681, 291118, 292366, 291960, 346490, 292187, 291318, 291321, 292189, 293756, 232710, 291322, 293327, 292194.

## Data Availability

Ithaca was trained on The Packard Humanities Institute’s Searchable Greek Inscriptions public dataset, PHI, which is available online (https://inscriptions.packhum.org/). The complete processing workflow for transforming the dataset to a machine-actionable format suitable for training Ithaca (I.PHI) is available at GitHub (https://github.com/sommerschield/iphi) under Apache License 2.0. The LGPN (https://www.lgpn.ox.ac.uk/) was used by annotators for the onomastics baseline to track the geographical and chronological distribution of ancient names. The PeriodO gazetteer (https://client.perio.do/) was used as a reference for mapping the PHI historical time periods to the chronological range metadata of I.PHI. The Pleiades gazetteer (https://pleiades.stoa.org/) was used as a reference for mapping the PHI region names to the geographical coordinates used in the geographical attribution map visualizations.

## Code availability

Ithaca’s training and inference source code is available at GitHub (https://github.com/deepmind/ithaca) under Apache License 2.0, along with the trained weights, licensed under Creative Commons Attribution-ShareAlike 4.0 International. A public interface for historians using Ithaca for their research (that is, restoration and attribution of Greek inscriptions, use of all visualization tools discussed in the present paper) is available online (https://ithaca.deepmind.com). Neural networks were developed with JAX v.0.2.9 (https://github.com/google/jax/), Flax v.0.3.0 (https://github.com/google/flax), and Haiku v.0.0.4 (https://github.com/deepmind/dm-haiku). The XLA compiler is bundled with JAX and does not have a separate version number. Dataset processing and analysis used Python v.3.7 (https://www.python.org/), NumPy v.1.19.2 (https://github.com/numpy/numpy), SciPy v.1.5.2 (https://www.scipy.org/), pandas v.1.1.3 (https://github.com/pandas-dev/pandas), beautifulsoup4 v.4.9.0 (https://www.crummy.com/software/BeautifulSoup/) and Google Colab (https://research.google.com/colaboratory), which is an online service and does not have a version number. Visualizations were generated using matplotlib v.3.4.2 (https://matplotlib.org/), seaborn v.0.11.1 (https://seaborn.pydata.org/) and GeoPandas v.0.9.0 (https://geopandas.org/).
