# Supplementary material for: Restoring and attributing ancient texts using deep neural networks
Source: Nature. 2022 Mar 9;603(7900):280–3. doi: 10.1038/s41586-022-04448-z (PMC8907065; doi:10.1038/s41586-022-04448-z)
Supplement: Supplementary file 1 — Reporting Summary [file 41586_2022_4448_MOESM1_ESM.pdf]

nature portfolio

Corresponding author(s): Yannis Assael, Thea Sommerschield

Last updated by author(s): Jan 19, 2022

Reporting Summary

Nature Portfolio wishes to improve the reproducibility of the work that we publish. This form provides structure for consistency and transparency in reporting. For further information on Nature Portfolio policies, see our Editorial Policies and the Editorial Policy Checklist.

Statistics

For all statistical analyses, confirm that the following items are present in the figure legend, table legend, main text, or Methods section.

n/a	Confirmed	
☐	☒	The exact sample size (n) for each experimental group/condition, given as a discrete number and unit of measurement
☐	☒	A statement on whether measurements were taken from distinct samples or whether the same sample was measured repeatedly
☒	☐	The statistical test(s) used AND whether they are one- or two-sided<br>*Only common tests should be described solely by name; describe more complex techniques in the Methods section.*
☒	☐	A description of all covariates tested
☐	☒	A description of any assumptions or corrections, such as tests of normality and adjustment for multiple comparisons
☐	☒	A full description of the statistical parameters including central tendency (e.g. means) or other basic estimates (e.g. regression coefficient) AND variation (e.g. standard deviation) or associated estimates of uncertainty (e.g. confidence intervals)
☒	☐	For null hypothesis testing, the test statistic (e.g. F, t, r) with confidence intervals, effect sizes, degrees of freedom and P value noted<br>*Give P values as exact values whenever suitable.*
☒	☐	For Bayesian analysis, information on the choice of priors and Markov chain Monte Carlo settings
☐	☒	For hierarchical and complex designs, identification of the appropriate level for tests and full reporting of outcomes
☒	☐	Estimates of effect sizes (e.g. Cohen's d, Pearson's r), indicating how they were calculated

Our web collection on statistics for biologists contains articles on many of the points above.

Software and code

Policy information about availability of computer code

Data collection	Ithaca's training and inference source code is available at https://github.com/deepmind/ithaca under Apache License 2.0, along with the trained weights, licensed under Creative Commons Attribution-ShareAlike 4.0 International (CC-BY 4.0). A public interface for historians using Ithaca for their research (i.e. restoration and attribution of Greek inscriptions, use of all visualization tools discussed in the present manuscript) is available at https://ithaca.deepmind.com.<br><br>Neural networks were developed with JAX v0.2.9 (https://github.com/google/jax/), Flax v0.3.0 (https://github.com/google/flax), and Haiku v0.0.4 (https://github.com/deepmind/dm-haiku). The XLA compiler is bundled with JAX and does not have a separate version number.
Data analysis	Dataset processing and analysis used Python v3.7 (https://www.python.org/), NumPy v1.19.2 (https://github.com/numpy/numpy), SciPy v1.5.2 (https://www.scipy.org/), pandas v1.1.3 (https://github.com/pandas-dev/pandas), beautifulsoup4 v4.9.0 (https://www.crummy.com/software/BeautifulSoup/), and Google Colab (https://research.google.com/colaboratory) which is an online service and does not have a version number.<br><br>Visualizations were generated using matplotlib v3.4.2 (https://matplotlib.org/), seaborn v0.11.1 (https://seaborn.pydata.org/), and GeoPandas v0.9.0 (https://geopandas.org/).

For manuscripts utilizing custom algorithms or software that are central to the research but not yet described in published literature, software must be made available to editors and reviewers. We strongly encourage code deposition in a community repository (e.g. GitHub). See the Nature Portfolio guidelines for submitting code & software for further information.

Data

Policy information about availability of data

All manuscripts must include a data availability statement. This statement should provide the following information, where applicable:

- Accession codes, unique identifiers, or web links for publicly available datasets
- A description of any restrictions on data availability
- For clinical datasets or third party data, please ensure that the statement adheres to our policy

Ithaca was trained on The Packard Humanities Institute's "Searchable Greek Inscriptions" public dataset, PHI, available at https://inscriptions.packhum.org/. The complete processing workflow for transforming the dataset to a machine-actionable format suitable for training Ithaca (I.PHI) is available at https://github.com/sommerschield/iphi under Apache License 2.0.

The Lexicon of Greek Person Names (LGPN) (https://www.lgpn.ox.ac.uk/) was used by annotators for the "Onomastics" baseline to track the geographical and chronological distribution of ancient names. The PeriodO gazetteer (https://client.perio.do/) was used as a reference for mapping the PHI historical time periods to the chronological range metadata of I.PHI. The Pleiades gazetteer (https://pleiades.stoa.org/) was used as a reference for mapping the PHI region names to the geographical coordinates used in the geographical attribution map visualizations.

Field-specific reporting

Please select the one below that is the best fit for your research. If you are not sure, read the appropriate sections before making your selection.

☒ Life sciences ☐ Behavioural & social sciences ☐ Ecological, evolutionary & environmental sciences

For a reference copy of the document with all sections, see nature.com/documents/nr-reporting-summary-flat.pdf

Life sciences study design

All studies must disclose on these points even when the disclosure is negative.

Sample size	For training the Ithaca model, no sample calculation was done, as the computational method proposed was evaluated on the full Packard Humanities Institute's "Searchable Greek Inscriptions" dataset. PHI is the largest public dataset of digitized ancient Greek inscriptions; when transforming it to a machine-actionable format, suitable for training Ithaca, it contains 78,608 inscriptions. Its large size allowed us to measure and demonstrate the effectiveness of our computational method in comparison to prior literature and the reported baselines.
Data exclusions	All inscriptions under 50 characters in length, excluding missing characters from the count, were removed; additionally, 9,441 duplicate texts were excluded.
Replication	To allow the replication of the results presented in this manuscript, Ithaca's training and inference source code is available at https://github.com/deepmind/ithaca under Apache License 2.0, along with a download link for trained weights, licensed under Creative Commons Attribution-ShareAlike 4.0 International (CC-BY 4.0). The processing workflow for transforming the Packard Humanities Institute's "Searchable Greek Inscriptions" public dataset to a machine-actionable format, suitable for training Ithaca, (I.PHI) is available at https://github.com/sommerschield/iphi, also under Apache License 2.0.
Randomization	Not applicable, we are not making a comparison between two groups.
Blinding	Not applicable, we are not making a comparison between two groups.

Reporting for specific materials, systems and methods

We require information from authors about some types of materials, experimental systems and methods used in many studies. Here, indicate whether each material, system or method listed is relevant to your study. If you are not sure if a list item applies to your research, read the appropriate section before selecting a response.

Materials & experimental systems

n/a	Involved in the study
☒	☐ Antibodies
☒	☐ Eukaryotic cell lines
☒	☐ Palaeontology and archaeology
☒	☐ Animals and other organisms
☒	☐ Human research participants
☒	☐ Clinical data
☒	☐ Dual use research of concern

Methods

n/a	Involved in the study
☒	☐ ChIP-seq
☒	☐ Flow cytometry
☒	☐ MRI-based neuroimaging
